# Phase I Trial of [^99m^Tc]Tc-maSSS-PEG_2_-RM26, a Bombesin Analogue Antagonistic to Gastrin-Releasing Peptide Receptors (GRPRs), for SPECT Imaging of GRPR Expression in Malignant Tumors

**DOI:** 10.3390/cancers15061631

**Published:** 2023-03-07

**Authors:** Vladimir Chernov, Anastasiya Rybina, Roman Zelchan, Anna Medvedeva, Olga Bragina, Nadejda Lushnikova, Artem Doroshenko, Evgeniy Usynin, Liubov Tashireva, Sergey Vtorushin, Ayman Abouzayed, Sara S. Rinne, Jens Sörensen, Vladimir Tolmachev, Anna Orlova

**Affiliations:** 1 Department of Nuclear Medicine, Cancer Research Institute, Tomsk National Research Medical Center, Russian Academy of Sciences, 634009 Tomsk, Russia; 2 Research Centrum for Oncotheranostics, Research School of Chemistry and Applied Biomedical Sciences, Tomsk Polytechnic University, 634050 Tomsk, Russia; 3 Department of General Oncology, Cancer Research Institute, Tomsk National Research Medical Center, Russian Academy of Sciences, 634009 Tomsk, Russia; 4 Department of General and Molecular Pathology, Cancer Research Institute, Tomsk National Research Medical Center, Russian Academy of Sciences, 634009 Tomsk, Russia; 5 The Laboratory of Molecular Therapy of Cancer, Cancer Research Institute, Tomsk National Research Medical Center, Russian Academy of Sciences, 634028 Tomsk, Russia; 6 Pathology Department, Siberian State Medical University, 634050 Tomsk, Russia; 7 Department of Medicinal Chemistry, Uppsala University, 751 23 Uppsala, Sweden; 8 Department of Surgical Sciences, Nuclear Medicine & PET, Uppsala University, 751 85 Uppsala, Sweden; 9 Department of Immunology, Genetics and Pathology, Uppsala University, 752 37 Uppsala, Sweden; 10 Science for Life Laboratory, Uppsala University, 752 37 Uppsala, Sweden

**Keywords:** GRPR, antagonist, 99mTc, phase I trial

## Abstract

**Simple Summary:**

Prostate and breast cancers are the most common malignancies. Accurate diagnosis and staging of diseases are important for the prognosis and determination of treatment tactics. The gastrin-releasing peptide receptor is overexpressed in over 80% of estrogen receptor-positive breast cancers and in up to 100% of primary prostate cancers, particularly in prostate cancers of lower grades and smaller sizes. Our group has developed an imaging agent [^99m^Tc]Tc-maSSS-PEG_2_-RM26 suitable for the detection of gastrin-releasing peptide receptors’ expression using SPECT. We aimed to perform a first-in-human study to test the safety of [^99m^Tc]Tc-maSSS-PEG_2_-RM26 administration, to study its biological distribution in normal organs, and to evaluate the agent’s targeting of receptors in tumors. This phase I study was performed in six prostate and seven breast cancer patients. Single injections of the new agent were well tolerated and a number of prostate and breast cancer primary tumors as well as metastases were visualized with SPECT/CT shortly after administration.

**Abstract:**

The gastrin-releasing peptide receptor (GRPR) is overexpressed in prostate cancer (PCa) and in hormone-driven breast cancer (BCa). The aim of this phase I clinical trial was to evaluate safety, biodistribution, and dosimetry after the administration of the recently developed GRPR-targeting antagonistic bombesin analogue [^99m^Tc]Tc-maSSS-PEG_2_-RM26 in PCa and BCa patients. Planar and whole-body SPECT/CT imaging was performed in six PCa patients and seven BCa patients 2, 4, 6, and 24 h post the intravenous administration of 40 µg of [^99m^Tc]Tc-maSSS-PEG_2_-RM26 (600–700 MBq). No adverse events or pathological changes were observed. The rapid blood clearance of [^99m^Tc]Tc-maSSS-PEG_2_-RM26 was observed with predominantly hepatobiliary excretion. The effective doses were 0.0053 ± 0.0007 for male patients and 0.008 ± 0.003 mSv/MBq for female patients. The accumulation of [^99m^Tc]Tc-maSSS-PEG_2_-RM26 in tumors was observed in four out of six PCa and in seven out of seven BCa patients. In four BCa patients, a high uptake of the agent into the axillary lymph nodes was detected. Immunohistochemistry revealed positive GRPR expression in 60% of primary PCa, 71.4% of BCa tumors, and 50% of examined BCa lymph nodes. In conclusion, a single administration of [^99m^Tc]Tc-maSSS-PEG_2_-RM26 was safe and well tolerated. [^99m^Tc]Tc-maSSS-PEG_2_-RM26 SPECT may be useful for tumor detection in PCa and BCa patients, pending further studies.

## 1. Introduction

Prostate and breast cancers are the most common sex-related malignancies, with an increasing incidence in many countries over the past 30 years [1]. Accurate diagnosis and staging of the diseases are important for the prognosis and determination of treatment tactics for these pathologies [2]. The detection and staging of prostate cancer (PCa) dramatically improved over the last decade due to wide introduction into the clinical practice of prostate-specific membrane antigen (PSMA)-targeting agents based on Glu-urea-Lys binding moiety. Many derivatives of this pseudo-peptide were tested clinically and the overall sensitivity of these agents was 70% in the detection of intra-prostatic cancer lesions, but only 61% in the detection of lymph node (LN) lesions (meta-analysis for 1256 patients [3]). Recently, two derivatives labeled with gallium-68 (Ga 68 PSMA-11) and with fluorine-18 (Piflufolastat F, 18, PYLARIFY^®^, also known as ^18^F-DCFPyL or PyL) were approved by the FDA for patients with suspected PCa metastasis and PCa recurrence after curative-intent therapy [4,5]. The PCa detection rate was similar for both imaging agents and their sensitivity positively correlated with patients’ PSA level [5,6]. However, both agents demonstrated limited sensitivity in the detection of LN metastases (61% for ^68^Ga-PSMA [3] and 40% for ^18^F-DCFPyL) [7]. This directly points to the fact that the diagnostic imaging of PCa requires improvement. The molecular targets associated with earlier stages of PCa, e.g., gastrin-releasing peptide receptors (GRPRs), could be utilized for the visualization of primary tumors, local recurrences, and extrapelvic LN metastatic lesions, potentially adding to diagnostic and staging accuracy.

The membrane-located GRPR is a G-protein coupled receptor. The overexpression of the GRPR in PCa reported in the literature varies from 100% [8,9] to 77% [10] of primary PCa. Over 85% of PCa LN metastases and castration-resistant PCa also express GRPRs; however, their expression level is lower [10]. GRPR expression is particularly associated with PCa of lower grade and smaller size, where PSMA imaging has demonstrated limited success [8,9]. The GRPR is also over-expressed in other cancers; gastrinomas (about 100%), colon (30–50%), renal, small cell lung, and uterine cancers overexpress GRPRs [11]. In breast cancer (BCa), the GRPR is mainly overexpressed in hormone-driven BCa [12]; the overexpression of GRPRs was found in 83.2% of estrogen receptor (ER)-positive BCa and only in 12% of ER-negative BCa, in 21.3% of human epidermal receptor type 2 (HER2)-positive tumors, and in 7.8% of triple-negative tumors [13].

Historically, the development of GRPR-targeting imaging agents was focused on agonistic bombesin (BN) peptide analogues [14]. However, the administration of agonists (even in trace amounts) results in adverse physiological response [15]. Additionally, the binding of agonists to GRPRs rapidly downregulates receptors’ membrane expression, resulting in a decreased number of targets available for the binding of tracers [16,17]. Therefore, current approaches in the development of imaging agents for the detection of GRPRs have shifted to the development of BN-based GRPR antagonists [14]. In phase I clinical study, the only adverse effect observed after the daily subcutaneous administration of GRPR-targeting therapeutic antagonist was local discomfort in the injection site at the highest studied doses (96 µg/kg) [18].

BN-based antagonistic peptides D-Phe-Gln-Trp-Ala-Val-Gly-His-Sta-Leu-NH_2_ (the derivatives of which are known as RM2, RM26, and BAY86-7548) and D-Phe-Gln-Trp-Ala-Val-Sar-His-Sta-Leu-NHEt (known as DB15) were successfully tested clinically for imaging GRPR expressions in PCa and BCa patients [19]. All clinical studies demonstrated the safety of the tracers, as well as the high-contrast imaging of GRPR expression in both types of cancers. The majority of tracers were developed for positron emission tomography (PET), i.e., the imaging technique that provides higher sensitivity than single-photon emission computer tomography (SPECT). The wider accessibility of SPECT scanners, recent progress in the development of the new generation of SPECT cameras, low costs of SPECT investigation, and the spread of PCa in the population all support the further development of GRPR imaging agents for SPECT. However, only one BN antagonist, [^99m^Tc]Tc-DB15, was tested in clinic so far, but in a limited number of patients (two cases) [20]. Our group has recently reported the development and pre-clinical evaluation of RM26 derivatives suitable for labeling with technetium-99m, i.e., [^99m^Tc]Tc-maSSS-PEG_2_-RM26 [21]. An amino-acid-based chelator (maSSS) at the N-terminus of the peptide is suitable for the rapid, easily performed, and stable attachment of technetium-99m to the peptide that should be beneficial for its production and clinical use.

Here, we report the results of a phase I clinical study on the use of [^99m^Tc]Tc-maSSS-PEG_2_-RM26.

The primary aims of this first-in-human study were (i) to test the tolerability and safety of an intravenous bolus injection of [^99m^Tc]Tc-maSSS-PEG_2_-RM26, (ii) to study the biological distribution of the labeled agent in normal organs at different time intervals, and (iii) to estimate the dosimetry for single imaging procedures using [^99m^Tc]Tc-maSSS-PEG_2_-RM26. Additionally, we aimed to evaluate the agent’s targeting of GRPRs in tumors by comparing SPECT imaging results and immunohistochemical (IHC) studies on patient-derived material in prostate and breast cancer.

## 2. Materials and Methods

### 2.1. Patients

**Figure 1:**
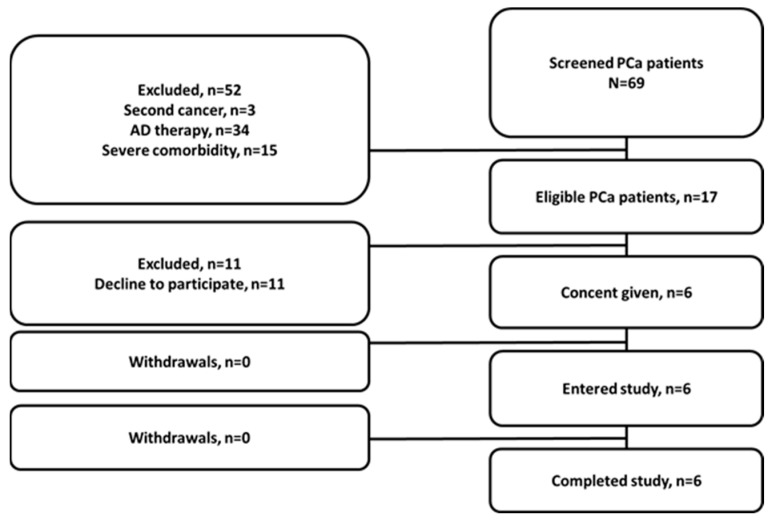
Flow diagram according to Standards for Reporting of Diagnostic Accuracy Studies for PCa patients.

**Figure 2:**
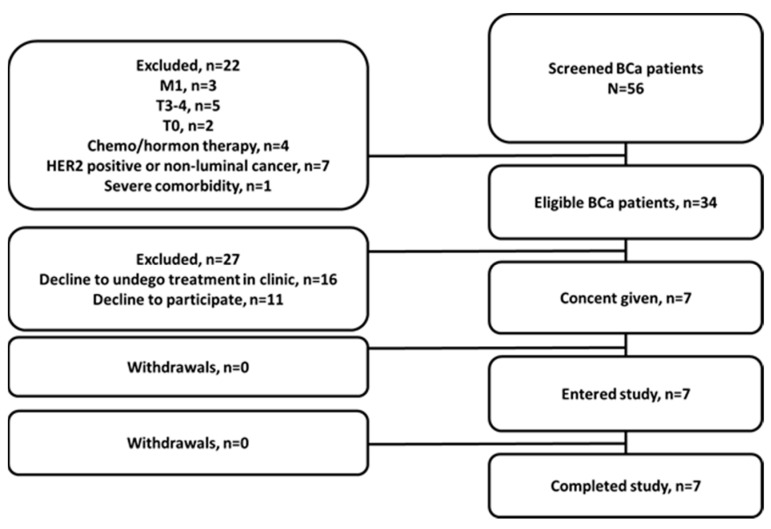
Flow diagram according to Standards for Reporting of Diagnostic Accuracy Studies for BCa patients.

A single-center diagnostic phase I open-label exploratory study (ClinicalTrials.gov ID NCT04746638) was conducted at the Cancer Research Institute, Tomsk National Research Medical Center of the Russian Academy of Sciences, and the initial protocol was approved by the Institute’s Scientific Council and Board of Medical Ethics (protocol 14, approved 21 December 2020). Patients (18–70 y) with clinically and radiologically diagnosed (with histological verification) PCa or BCa were included in this study after providing written informed consent. Criteria for inclusion were: the ability to undergo planned diagnostic investigations; patient’s hematological, liver, and renal function test results within the normal limits; and a negative pregnancy test for BCa patients. The exclusion criteria included contraindications to surgical intervention due to severe concomitant pathology; a second malignancy (non-breast or non-prostate); activity/history of autoimmune disease, hepatitis B or C, HIV, or infectious diseases within the preceding 3 months; participation in other clinical studies; and ongoing toxicity over grade 2 from previous standards or investigational therapies. Additionally, for PCa patients, exclusion criteria were androgen deprivation therapy over 3 months, and for BCa patients, exclusion criteria were HER2-positive or non-luminal cancer, in situ T0 and T3-4 tumors, the presence of distant metastases, and history of chemo- and/or hormone-therapy. Six male PCa and seven female BCa patients were included in the study (Figure 1 and Figure 2).

As a local standard of care, patients with BCa underwent standard mammography (GIOTTO IMAGE, IMS SRL), ultrasound imaging of the breast, regional lymphatic nodes and liver (LOGIQ E9, GE), and biopsy sampling of primary tumors with a histological and immunohistochemical (IHC) (ER, PR, HER2 by Ventana) examination. Fluorescent in situ hybridization analysis was performed in two patients. An examination of patients with PCa included a measurement of prostate-specific antigen (PSA) levels, digital rectal examination, transrectal ultrasound (HI VISION AVIUS, Hitachi Aloka, Twinsburg, OH, USA), an MRI of the pelvic organs with contrast (Essenza, Siemens, Munich, Germany), and a prostate biopsy with histological examination. Patients of both groups underwent chest CT and bone scans using ^99m^Tc-pyrophosphate. LN metastases before treatment were confirmed by a histological (using core biopsy) or cytological (using fine-needle biopsy) examination in all breast cancer patients.

In all cases of BCa and PCa, surgery with a morphological assessment of the removed tumor and LN was performed.

### 2.2. Imaging Protocol

Kits containing maSSS-PEG_2_-RM26 were used for labeling with technetium-99m eluate according to the previously reported protocol [21]. More details on kit formulation will be published later elsewhere. The radiochemical purity was over 98%.

Patients were injected with [^99m^Tc]Tc-maSSS-PEG_2_-RM26 (40 µg of maSSS-PEG2-RM26 labeled with 640 ± 165 MBq for PCa and 695 ± 120 MBq for BCa patients per injection) as a single intravenous bolus. Imaging was performed using a hybrid system (Symbia Intevo T16), equipped with a dual-head gamma camera and an integrated 16-slice CT scanner. For imaging, a low-energy high-resolution collimator was used. Patients underwent whole-body planar images (anterior and posterior, with a of scan speed 12 cm/min in a 1024 × 256 pixel matrix) and SPECT-CT acquisition (32 projections, 30 s each, in a 128 × 128 pixel matrix) and a low-dose CT (140 kVp, 20 mAs/slice in a 512 × 512 pixel matrix) were performed 2, 4, 6, and 24 h after injection. The images were transferred to a Syngo.via (Siemens) workstation for analysis.

Blood and urine were analyzed before injection and 1 d after. Vital signs (blood pressure, pulse, respiratory rate, and temperature and ECG) were monitored before and within 24 h pi of imaging probe, and an evaluation of the possible side effects was performed within 3–7 d after injection.

### 2.3. Immunohistochemical Evaluation of Patient Material on GRPR Expression

GRPR expression was assessed by immunohistochemistry using anti-GRPR rabbit polyclonal primary antibody (1:500, Thermo Fisher Scientific, Waltham, MA, USA) in resected tumours and LN in patients without systemic therapy or biopsy tumour samples before chemotherapy. The largest LN from all dissected was taken for IHC analysis. Thus, we are not certain that the LN investigated by the IHC was detected by [^99m^Tc]Tc-maSSS-PEG_2_-RM26 imaging. Tissue sections (4 µm, microtome RM2255, Thermo Fisher Scientific, Waltham, MA, USA) were mounted to X-tra adhesive slides (Leica, Wetzlar, Germany). Samples were stained on the Ventana BenchMark Ultra platform (Roche, Oro Valley, AZ, USA) using CC1 standard antigen retrieval and the enhanced OptiView detection system. In all samples, GRPR expression was evaluated as follows. The percentage of positively stained tumour cells was estimated and staining intensity was subjectively assessed in malignant epithelial tissue according to a four-point scoring system comprising scores of 0 (negative), 1 (+), 2 (++), or 3 (+++).

### 2.4. Analysis of Biodistribution and Assessment of Dosimetry

The geometric means of counts in the regions of interest (ROIs) drawn over organs and the whole body for anterior and posterior projections were calculated for every time point. An ROI for the heart was used to estimate activity concentrations in the blood. Quantification was performed as described previously [22]. Absorbed doses for individual organs, effective doses, and effective dose equivalents were calculated in OLINDA/EXM 1.1 (adult male phantom for PCa and adult female phantom for BCa) using average residence times derived from activity distributions over time fitted to a single exponential using Prism 9.2.0 (GraphPad Software, LLC, Boston, MA, USA).

### 2.5. Statistical Analysis

Data are reported as means with standard deviations (n = 6 for PCa and n = 7 for BCa). The median and interquartile range Me [Q1–Q3] were used for presenting nonparametric data. Differences of significance (one-way ANOVA, two-side, *p* < 0.05) were tested using Prism 9.2.0.

## 3. Results

### 3.1. Patients

In total, 13 patients were recruited into this study: recently diagnosed 6 males with PCa and 7 females with breast cancer (Table 1 and Table 2). For PCa patients, the median PSA level was 10.1 [7.93–207.6].

### 3.2. Safety, Tolerability, and Distribution of [^99m^Tc]Tc-maSSS-PEG_2_-RM26

Both male and female patients tolerated injections well. No adverse events, pathological changes in clinical laboratory tests, or changes in vital signs were observed after a single intravenous bolus injection of 40 µg of [^99m^Tc]Tc-maSSS-PEG2-RM26.

**Figure 3:**
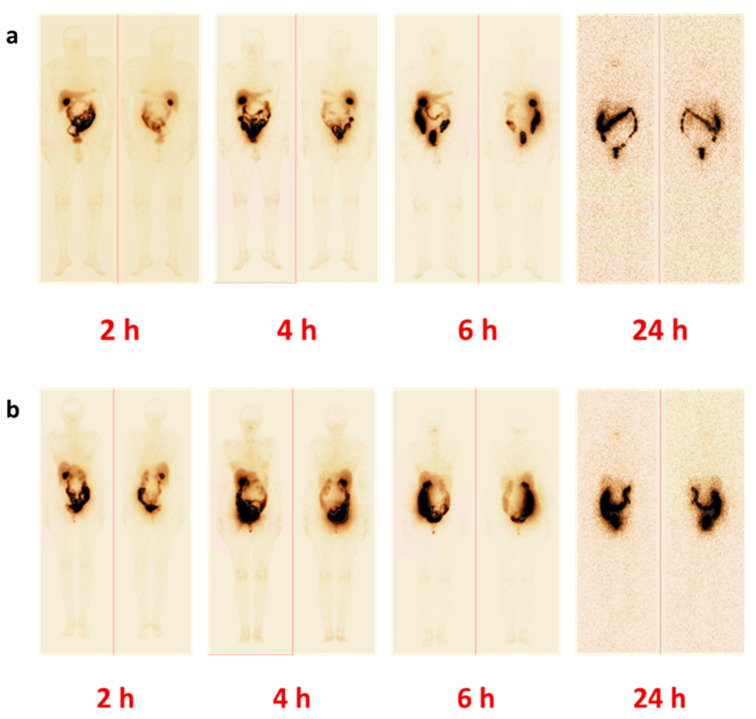
Representative anterior and posterior images of patients with (**a**) PCa (P2) and (**b**) BCa (B6) 2, 4, 6, and 24 h pi of [^99m^Tc]Tc-maSSS-PEG_2_-RM26.

The whole body distribution of [^99m^Tc]Tc-maSSS-PEG2-RM26 in PCa and BCa patients over time is presented in Figure 3 and Table A1. While overall activity distribution pattern was similar for both cohorts, the activity uptake in healthy organs and tissues was higher in female patients.

**Figure 4:**
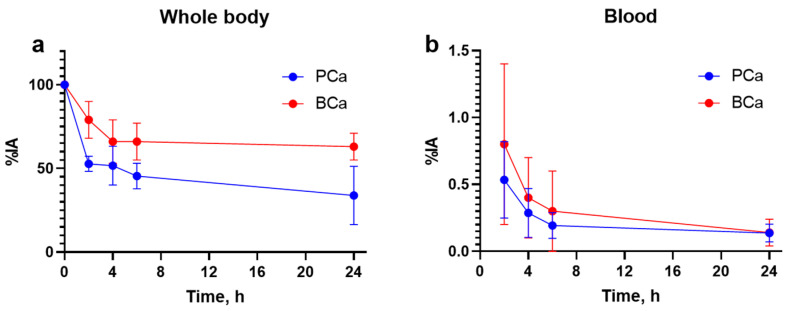
Kinetics of whole body elimination (**a**) and blood (**b**) clearance of [^99m^Tc]Tc-maSSS-PEG_2_-RM26.

Rapid whole body elimination of [^99m^Tc]Tc-maSSS-PEG_2_-RM26 was observed in both cohorts, however general elimination was significantly more efficient in male patients: T_½_ = 1.14 h for male (R^2^ = 0.95) and T_½_ = 1.48 h (R^2^ = 0.99) for female patients (Figure 4a). Clearance from blood circulation was similar for both cohorts (T_½_ elimination half-life of 1.6 ± 0.2 h for male patients and 1.5 ± 0.2 h for female patients (Figure 4b); however, the passage of activity through the gastrointestinal tract was more efficient in male patients according to the results of whole body imaging (Figure 3).

The elimination of [^99m^Tc]Tc-maSSS-PEG_2_-RM26 took place both via both renal and hepatobiliary pathways (Table A1). Initially, elevated activity concentrations in urine observed 2 h pi considerably decreased with time and 4 h pi was two-fold lower for male and four-fold lower for female patients. Prominent activity (over 3% of injected activity) was observed also in liver and kidneys 2 h pi; however, the hepatic uptake decreased with time, while the renal one did not. Up to 40% of injected activity was excreted via bile and elevated activity uptake was observed in organs of gastrointestinal tract: gall bladder, jejunum, as well as upper and lower colon. Accordingly, the highest activity was initially found in gall bladder and small intestines. Starting from 4 h pi injection, high activity levels were observed in the upper large intestine.

### 3.3. Assessment of Dosimetry

An evaluation of the absorbed doses was performed based on whole body images at 2, 4, 6, and 24 h pi, as shown in Table 3. Usually, absorbed doses in healthy organs and tissues after administration of [^99m^Tc]Tc-maSSS-PEG_2_-RM26 did not differ significantly for male and female patients. However, absorbed doses for pancreas and liver were significantly higher (*p* < 0.05) for female patients. The same trend was found for organs of gastrointestinal tract (significantly higher for gall bladder wall, *p* < 0.05). The absorbed dose for kidneys was higher for male patients. The effective doses were 0.0053 ± 0.0007 for male and 0.008 ± 0.003 mSv/MBq for female patients with the given activity. The radionuclide-associated dose burden per patient was 3-6 mSv/study.

### 3.4. Imaging Data Analysis

**Figure 5:**
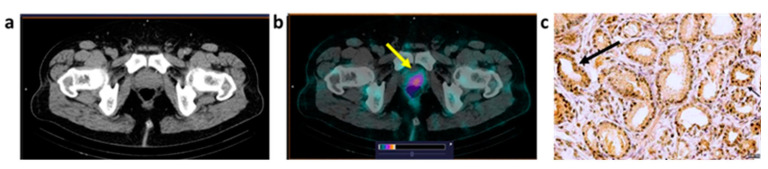
CT (**a**) and fused SPECT/CT (**b**) images of PCa patient (P1, adenocarcinoma) 2 h after injection of [^99m^Tc]Tc-maSSS-PEG_2_-RM26. A focus of increased [^99m^Tc]Tc-maSSS-PEG_2_-RM26 uptake (SUVmax = 1.21) is visualized in the prostate (yellow arrow). The upper setting of the scale window (24% of the maximum number) was adjusted to visualize the lesion. Moderate (2+) GRPR expression in adenocarcinoma cells (**c**) was detected by IHC analysis in tumor material (black arrow). Magnification 40×.

**Figure 6:**
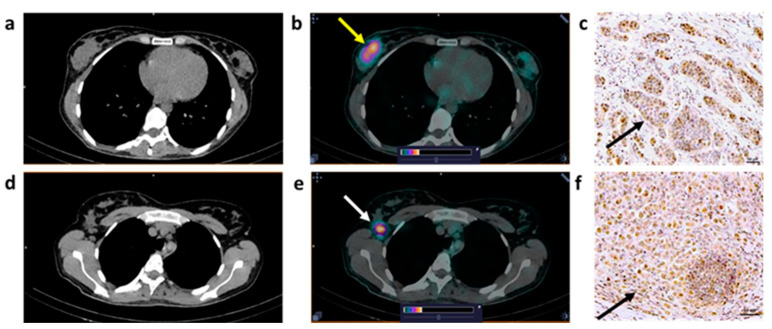
CT (**a**,**d**) and fused SPECT/CT (**b**,**e**) images of BCa patient (B2, invasive carcinoma) 2 h after injection of [^99m^Tc]Tc-maSSS-PEG_2_-RM26. A focus of increased [^99m^Tc]Tc-maSSS-PEG_2_-RM26 uptake (SUVmax = 1.75) is visualized in the right breast ((**b**), yellow arrow). An enlarged (up to 1.3 cm) right axillary node with elevated [^99m^Tc]Tc-maSSS-PEG_2_-RM26 uptake (SUVmax = 1.8) is visualized ((**e**), white arrow).The upper setting of the scale window (24% of the maximum number) was adjusted to visualize the lesion. Light (1+) GRPR expression and moderate (2+) expression (black arrow) in carcinoma cells were detected by IHC analysis in primary BCa tumor (**c**) and in LN (**f**). Magnification 40×.

Representative images of PCa and BCa lesions 2 h pi of [^99m^Tc]Tc-maSSS-PEG_2_-RM26 are provided in Figure 5 and Figure 6. All tumors and metastases with high [^99m^Tc]Tc-maSSS-PEG_2_-RM26 uptake were clearly visualized already 2 h after injection.

The uptake of [^99m^Tc]Tc-maSSS-PEG_2_-RM26 in tumors was identified in four out of six PCa patients. According to CT measurements, the median size of visualized PCa tumors was 3.22 cm^3^ [2.9–16.1]. In two PCa patients, [^99m^Tc]Tc-maSSS-PEG_2_-RM26 uptake in the tumor was not visualized.

The increased accumulation of [^99m^Tc]Tc-maSSS-PEG_2_-RM26 in the tumor was observed in seven out of seven BCa patients. According to CT measurements, the median size of visualized BCa tumors was 13.6 cm^3^ [1.6–73.9]. In four BCa patients, a high uptake was also detected in the axillary LN. According to CT measurements, the median size of visualized axillary LN was 3.72 cm^3^ [1.1–7.8]. Interestingly, in two patients (B3 and B4), no signs of regional metastases were detected by standard methods of examination, but a pathological accumulation of [^99m^Tc]Tc-maSSS-PEG_2_-RM26 in the axillary was detected on SPECT/CT. Subsequently, these patients underwent surgical treatment with the determination of sentinel LN. Metastases in the LN were detected by morphological examination during surgery, as a result of which the operation was expanded to a standard axillary lymphadenectomy. The stage of the disease according to postsurgical morphological results changed in patient B3 from N0 to N3 and in patient B4 from N0 to N1 (Table 1).

In different clinical cases, results of SPECT/CT confirmed LN metastases in B2 patient which were detected based on the results of clinical, instrumental, and cytological examinations. After axillary lymphadenectomy, metastases were found in all 26 removed LNs according to the postsurgical morphological results and the stage of the disease changed.

Two hours after the injection of [^99m^Tc]Tc-maSSS-PEG_2_-RM26, the median (Me [Q1–Q3]) activity accumulation (SUVmax) was 1.18 [0.78–1.67] in prostate tumors, 0.87 [0.43–1.75] in breast tumors, and 1.8 [0.58–1.8] in LN metastases in BCa patients. The median tumor/background value was 6.85 [4.2–10.9] for prostate tumors, 7.8 [2.2–35] for breast tumors, and 9.2 [6–16.3] for LN metastases. With time, activity accumulation and tumor/background values decreased in all lesions.

### 3.5. Immunohistochemical Staining

The immunohistochemical staining results show positive GRPR expressions in three out of five tumor samples in PCa patients (60%) and in five of seven tumor tissue in BCa patients (71.4%). LNs were examined in four BCa patients who had LN metastasis and 50% (2/4) patients showed positive GRPR expressions in examined LNs. It was not possible to assess the relationship with clinical parameters or correlations with [^99m^Tc]Tc-maSSS-PEG_2_-RM26 accumulation because of the small sample size.

## 4. Discussion

The introduction of PSMA-specific probes was a revolution in the radionuclide imaging of PCa. However, the accumulation of clinical information helped researchers understand that the sensitivity of imaging should be further improved [3]. In this study, we performed an initial (Phase I) clinical assessment of a tracer, which should visualize an additional target in PCa, known as the GRPR. This tracer may be used as a complement to PSMA imaging probes in PCa. In addition, there are indications for the use of this tracer for the identification of ER-positive BCa and consequent treatment in hormone therapy.

The results of this study suggest that 40 µg injections of [^99m^Tc]Tc-maSSS-PEG_2_-RM26 had no observable side effects, i.e., they were safe and tolerable. Importantly, no event could be linked to the agonistic signaling of the labelled compound. This is essential because the injected peptide mass in this study was two orders of magnitude higher than the injected mass of agonistic bombesin derivative, which was reported to induce adverse side effects [23]. On the other hand, the absence of the agonistic action-related side effects is in good agreement with the data concerning an evaluation of other imaging probes based on GRPR antagonists, which were injected within the same mass range, i.e., 10–56 µg/injection [20,24,25]. The absence of any pharmacological effect from GRPR antagonists should improve the perception of the imaging procedure by patients and help to avoid side-effect-related artifacts. This allows to optimize the injected peptide mass that could improve imaging sensitivity (see below).

The biodistribution of [^99m^Tc]Tc-maSSS-PEG_2_-RM26 was characterized by the rapid whole body elimination and blood clearance of unbound tracers (Figure 4). This decreases the background caused by the blood-borne activity and creates preconditions for high-contrast imaging. The clinical trial confirmed the major feature of the tracer found in the preclinical studies, a substantial degree of hepatobiliary excretion [21]. The hepatobiliary excretion of [^99m^Tc]Tc-maSSS-PEG_2_-RM26 was the highest among other GRPR antagonists tested in clinics and was similar to the excretion of [^99m^Tc]Tc-RP527, a GRPR agonist [26]. Predominantly renal excretion was observed for [^68^Ga]Ga-BAY-7548 [27], [^64^Cu]Cu-CB-TEA2-AR06 [25], [^68^Ga]Ga-SB3 [28], [^68^Ga]Ga/[^177^Lu]Lu-RM2 [24,29], [^68^Ga]Ga-NeoBOMB1 [30], and [^99m^Tc]Tc-DB15 [20]. Up to 60-70% of injected activity for these short peptides is excreted to urine within 4 h [31] and the reported images for these compounds reveal appreciable activity uptake in the urinary bladder. Previously, we developed an imaging probe [^68^Ga]Ga-PEG_3_-RM26 to visualize GRPR expression using PET [32,33], which also had renal excretion. In the present study, we tested an imaging probe with predominantly hepatobiliary excretion. This feature was designed to enable the removal of interfering activity in blood and also prevent the accumulation of high activity in the urinary bladder, which would be unfavorable for the imaging of primary PCa and its local LN metastases. This strategy was used also in the development of ^18^F-labeled fluorocholine [34]. However, the activity excreted enterohepatically may worsen the visualization of lesions in the lower abdomen at later time points and may complicate the visualization of hepatic metastases, thus limiting the clinical application of this imaging agent. Still, the formation of hepatic metastases in PCa is a relatively late event, as opposite to BCa, where the liver is a site of early metastatic spread. It is interesting to note that male patients had a somewhat more rapid passage of enterohepatically excreted activity in the gastrointestinal tract than female patients. A similar phenomenon was also observed in the studies with a radiolabeled GRPR agonist [26]. This phenomenon could reflect physiological differences between genders, and should be taken in account in the development of imaging agents. The longer residence time for activity accumulated in the organs of the GI tract resulted in higher absorbed doses for organs in the abdomen (liver) and organs in the GI tract (pancreas and kidneys).

In this the first phase I study, we demonstrated that the use of [^99m^Tc]Tc-maSSS-PEG_2_-RM26 resulted in effective doses of 0.0053 ± 0.0007 mSv/MBq for PCa and 0.008 ± 0.003 mSv/MBq for BCa. Unfortunately, effective doses for GRPR antagonists were not published yet [20], but effective doses for [^99m^Tc]Tc-maSSS-PEG_2_-RM26 were found to be similar to doses reported for ^99m^Tc-labeled GRPR agonistic imaging probes (0.009 ± 0.001 mSv/MBq for mixed cohorts of PCa and BCa patients [26] and 0.005 ± 0.002 mSv/MBq for PCa patients) [35]. It is appreciably lower than effective doses after the injection of antagonists ^64^Cu-CB-TE2A-AR06 (0.019 ± 0.008 mSv/MBq [25]), ^68^Ga-SB3 (0.0144 mSv/MBq [36]), and ^68^Ga-RM26 (0.07 ± 0.01 mSv/MBq [33]) because of the absence of particle emission.

Several clinical trials on the PET imaging of GRPR expression using antagonistic peptides were reported in the past decade [19]. It has to be noted that while PET provides better imaging resolution, SPECT with technetium-99m-labeled probes offers wider availability, as well as lower costs of imaging facilities and imaging agents. Furthermore, SPECT spatial resolution and sensitivity both significantly improved in recent cameras with cadmium–zinc–telluride (CZT) detectors and their quantification accuracy is comparable to PET/CT [37]. The count rate per injected activity of SPECT cameras remains lower than the rate of PET, but lower absorbed and equivalent doses permit injections of larger activities of ^99m^Tc-labeled tracers. However, so far, only one pilot proof-of-principle study (in two patients with advanced BCa) concerning the imaging of GRPR expression using the GRPR antagonist [^99m^Tc]Tc-DB15 was reported [20]. The current study provides another evaluation of this class of tracers, contributing to its further development.

In this phase I study, patients with recently diagnosed PCa or BCa cancer were recruited. Four of six primary PCa lesions were visualized on SPECT/CT after the administration of [^99m^Tc]Tc-maSSS-PEG_2_-RM26, with the smallest being 2.9 cm^3^. In visualized primary PCa tumors, a reasonable contrast was achieved, the highest one was observed for the patient with the biggest tumor and the highest PSA value. In BCa patients, all primary tumors were clearly visualized, with the smallest being 1.6 cm^3^. In addition, LN metastases were visualized in three BCa patients, with the smallest being 1.1 cm^3^. It should be noted that it is not certain that the LN investigated by IHC analysis has been detected by [^99m^Tc]Tc-maSSS-PEG_2_-RM26 imaging due to the study design. The study protocol for phase II should be adjusted for better matching. Although the determination of sensitivity and specificity is not a goal of phase I studies, the imaging data are of interest because they may help to design further investigations. In this study, no clear correlation between a level of GRPR expression and SUVmax in tumors was found (Table 1 and Table 2). Apparently, the small number of patients does not allow a strict statistical analysis. However, this may be an indication that the further optimization of imaging protocols is required. An essential optimization parameter may be an injected mass of [^99m^Tc]Tc-maSSS-PEG_2_-RM26. It was demonstrated for the somatostatin analogue, [^111^In]-octreoscan, demonstrated that the tumor uptake has a bell-shaped dependence on the injected mass, and injection of a mass that is too low (below 5 µg) may be associated with false-negative clinical findings [38,39]. Similarly, bell-shaped dependence was also found for both agonistic [40] and antagonistic [41] analogues of bombesin. Clinical studies with radiolabeled scaffold proteins demonstrated that the injected protein mass is an essential parameter [22,42,43] and the maximum sensitivity and specificity may be achieved only in a relatively narrow range of injected doses. Thus, future phase II studies should evaluate the role of injected peptides mass in providing imaging with sufficient sensitivity and specificity. It has to be noted that even with the current non-optimized imaging protocol, the use of [^99m^Tc]Tc-maSSS-PEG_2_-RM26 enabled diagnosis of LN involvement in two cases of BCa (one unknown lesion was as small as 1.4 cm^3^), not detected by standard investigations.

## 5. Conclusions

Single injection of the antagonistic GRPR-targeting peptide-based imaging probe [^99m^Tc]Tc-maSSS-PEG_2_-RM26 is safe. Single injections were well tolerated and associated with low absorbed doses to healthy organs and tissue. The effective dose was similar to the doses from other imaging probes of the same class. A number of PCa and BCa primary tumors, as well as BCa LN metastases, were visualized with SPECT/CT shortly after administration. Further clinical studies are justified to evaluate the sensitivity and specificity of GRPR imaging using [^99m^Tc]Tc-maSSS-PEG_2_-RM26.

## 6. Patents

Patent 2,776,234 C1 (14.07.2022, application № 2021124249 12.08.2021)), Russian Federation.

## Figures and Tables

**Table 1:** PCa patient characteristics.

Patient No	Age, y	Histotype PSA ^1^	Clinical Stage	SUVmax (Lesion Size, cm) ^2^	Ratio SUVmax in Tumor to Background	IHC GRPR Status ^3^
P1	68	PAA G2 GS 7 (3+4) PSA = 11.4	T2N0M0	1.67 (1.6 × 2.5)	7.3	2+
P2	56	PAA G2 GS 7 (3+4) PSA = 9	T2aN0M0	0.78 (1.7 × 2)	6.5	0
P3	68	PAA G5 GS 9 (4+5) PSA = 207.6	T3aN0M0	1.21 (4.1 × 2.8)	10.9	No material
P4	65	PAA G1 GS 6 (3+3) PSA = 9.25	T1bN0M0	1.14 (1.8 × 2.0)	4.2	1+
P5	66	PAA G1 GS 6 (3+3) PSA = 11	T2N0M0	No accumulation		3+
P6	70	PAA G2 GS 7 (4+3) PSA = 7.93	T2aN0M0	No accumulation		0

^1^ Histological diagnosis: PAA—prostatic acinar adenocarcinoma. G—(ISUP grade group). GS—Gleason score. PSA—prostate-specific antigen concentration in blood, ng/mL. ^2^ Two hours post administration, lesion size was obtained based on SPECT/CT. ^3^ 0—negative, 1+—light, 2+—moderate, 3+—strong.

**Table 2:** BCa patient characteristics.

Patient No	Age, y	Histotype ^1^	Clinical Stage	SUVmax (Lesion Size. cm) ^2^	Ratio SUVmax in Tumor to Background	IHC GRPR Status ^3^	Pathologic Stage
B1	34	IC NST G2	T2N0M0	T: 1.41 (2.8 × 2.2)	2.2	T: 3+	T2N0M0
B2	41	IC NST G2	T2N2M0	T: 1.75 (3.8 × 2.2) LNM: 1.8 (1.3)	T: 35 LNM: 16.3	T: 1+ LNM: 2+	T2N3M0
B3	40	IC NST G2	T2N0M0	T: 0.87 (5.1 × 2.2) LNM: 1.8 (1.4)	T: 10.8 LNM: 7.1	T: 0 LNM:1+	T3N3M0
B4	69	ILC G1	T2N0M0	T: 0.57 (6.8 × 3.2) LNM: 0.58 (2.5)	T: 7.8 LNM: 6	T: 1+ LNM: 1+	T2N1M0
B5	56	IC NST G2	T2N1M0	T: 0.87 (4.0 × 1.7) LNM: 1.8 (2.3)	T: 14.5 LNM: 11.3	T: 0 LNM: 0	T2N1M0
B6	50	IC NST G1	T1N0M0	T: 0.43 (1.5 × 1.5)	T: 4.3	T: 3+	T1N0M0
B7	62	IC NST G2	T1N0M0	T: 0.50 (1.5 × 1.4)	T: 2.7	T: 1+	T1N0M0

^1^ Histological diagnosis: IC NST—invasive carcinoma of no special type; ILC—invasive lobular carcinoma. ^2^ Two hours post administration, lesion size was obtained based on SPECT/CT; T—tumor, LNM—single LN metastasis. ^3^ 0—negative, 1+—light, 2+—moderate, 3+—strong.

**Table 3:** Absorbed doses after the iv injection of [^99m^Tc]Tc-maSSS-PEG_2_-RM26.

Organ	Males (n = 6)	Females (n = 7)
Adrenals	0.005 ± 0.002	0.006 ± 0.001
Brain	0.0006 ± 0.0002	0.0005 ± 0.0002
Breasts	0.0006 ± 0.0002	0.0007 ± 0.0002
Gallbladder wall	0.011 ± 0.004	0.06 ± 0.03 ^1^
Lower large intestine wall	0.008 ± 0.001	0.013 ± 0.007
Small intestine	0.014 ± 0.005	0.03 ± 0.02
Stomach wall	0.004 ± 0.001	0.005 ± 0.002
Upper large intestine wall	0.05 ± 0.02	0.04 ± 0.03
Heart wall	0.0021 ± 0.0006	0.0026 ± 0.0009
Kidneys	0.014 ± 0.007	0.012 ± 0.004
Liver	0.004 ± 0.001	0.0074 ± 0.0016 ^1^
Lungs	0.0015 ± 0.0003	0.0018 ± 0.0005
Muscle	0.0018 ± 0.0006	0.0022 ± 0.0006
Ovaries	N/A ^2^	0.018 ± 0.008
Pancreas	0.003 ± 0.001	0.0058 ± 0.0005 ^1^
Red marrow	0.0026 ± 0.0009	0.0030 ± 0.0009
Osteogenic cells	0.005 ± 0.002	0.005 ± 0.001
Skin	0.0011 ± 0.0005	0.0010 ± 0.0003
Spleen	0.004 ± 0.002	0.005 ± 0.001
Testes	0.004 ± 0.005	N/A
Thymus	0.0017 ± 0.0005	0.0023 ± 0.0010
Thyroid	0.0014 ± 0.0005	0.0016 ± 0.0009
Urinary bladder wall	0.006 ± 0.004	0.008 ± 0.006
Uterus	N/A	0.020 ± 0.008
Total body	0.0026 ± 0.0009	0.0031 ± 0.0009
Effective dose equivalent (mSv/MBq)	0.009 ± 0.002	0.015 ± 0.003 ^1^
Effective dose (mSv/MBq)	0.0053 ± 0.0007	0.008 ± 0.003

^1^ Dose was significantly higher for females than for males (*p* < 0.05). ^2^ N/A—not applicable.

## Data Availability

The data generated during the current study are available from the corresponding author upon reasonable request.

## Appendix A

**Table A1:** Distribution of [^99m^Tc]Tc-maSSS-PEG2-RM26 in healthy organs and tissues. Data are presented as % ID/organ and standard deviation.

**Male/PCa**	**2 h**	**4 h**	**6 h**	**24 h**
Kidney	2.9 ± 0.9	3 ± 3	3 ± 1	2.4 ± 0.9
Urinary bladder	4 ± 6	2 ± 1	0.6 ± 0.4	0.16 ± 0.07
Liver	4 ± 2	2.2 ± 0.8	1.8 ± 0.6	2 ± 1
Gall bladder	2 ± 2	2 ± 2	2 ± 2	2.2 ± 0.3
Jejunum	12 ± 12	9 ± 9	9 ± 19	10 ± 1
Upper colon	1.1 ± 1.0	8 ± 3	20 ± 13	7 ± 8
Lower colon	0.6 ± 0.5	0.2 ± 0.2	0.5 ± 0.4	0.4 ± 0.2
**Female/BCa**	**2 h**	**4 h**	**6 h**	**24 h**
Kidney	5 ± 3	3 ± 3	3 ± 3	5 ± 5
Urinary bladder	4 ± 7	1 ± 2	0.3 ± 0.3	0.2 ± 0.2
Liver	6 ± 2	3.0 ± 1.0	3 ± 2	2 ± 2
Gall bladder	5 ± 5	5 ± 4	4 ± 3	0.3 ± 0.1
Jejunum	21 ± 9	25 ± 17	13 ± 10	3 ± 2
Upper colon	1.1 ± 0.6	6 ± 12	19 ± 17	15 ± 10
Lower colon	1.4 ± 0.8	1.0 ± 0.9	0.8 ± 0.7	20 ± 15
